# Downregulation of Tim-1 inhibits the proliferation, migration and invasion of glioblastoma cells via the miR-133a/TGFBR1 axis and the restriction of Wnt/β-catenin pathway

**DOI:** 10.1186/s12935-021-02036-1

**Published:** 2021-07-05

**Authors:** Li Wei, Ya Peng, Naiyuan Shao, Peng Zhou

**Affiliations:** 1 Department of Tumor Biological Treatment, The Third Affiliated Hospital of Soochow University, Changzhou, Jiangsu 213003 China; 2 Department of Neurosurgery, The Third Affiliated Hospital of Soochow University, No. 185 Bureau Front Street, Changzhou, 213003 Jiangsu China

**Keywords:** Glioblastoma, T-cell immunoglobulin and mucin domain 1, MicroRNA-133a/TGFBR1 axis, Proliferation, Invasion, Migration

## Abstract

**Background:**

Glioblastoma remains one of the most lethal brain cancers. T-cell immunoglobulin and mucin domain 1 (Tim-1) is associated with various immune diseases. The molecular mechanism of Tim-1 in regulating glioblastoma cell proliferation, invasion, and migration is still unknown. Moreover, it has shown that miR-133a plays an important role in glioblastoma. However, little is known about the interaction between Tim-1 and miR-133a in glioblastoma.

**Methods:**

Tim-1 expression in glioblastoma and normal brain tissues was detected by qPCR, Western Blot and IHC. After Tim-1 knockdown in U251 and U87 cells, genes showing significantly differential expression, along with the significant differential miRNAs were analyzed using RNA-seq analysis. The binding sites were verified using dual-luciferase reporter gene assay. U251 and U87 cells were allocated into the small harpin-negative control (sh-NC), sh-Tim-1, sh-Tim-1 + inhibitor NC, and sh-Tim-1 + miR-133a inhibitor group. Cell proliferation, migration, and invasion were determined by CCK-8, flow cytometry, wound-healing and Transwell assays, respectively. Next, U251 and U87 cells were allocated into the mimic NC, miR-133a mimic, miR-133a mimic + pcDNA3.1, and miR-133a mimic + pcDNA3.1-TGFBR1 groups, followed by the detection of cell proliferation, migration, and invasion. Western blot was used to identify the expression of vital kinases in the Wnt/β-catenin pathway.

**Results:**

Tim-1 was highly expressed in glioblastoma tissues compared with that in normal brain tissues. RNA-seq analysis showed that Tim-1 knockdown could lead to the downregulation of TGFBR1 and the upregulation of miR-133a. The binding sites between TGFBR1 and miR-133a were confirmed. Tim-1 knockdown impaired the invasion, migration, proliferation of U251 and U87 cells, which could be reversed by miR-133a downregulation. miR-133a upregulation inhibited the proliferation, invasion, and migration of U251 and U87 cells, which could be reversed by TGFBR1 upregulation. Tim-1 knockdown and miR-133a upregulation could inhibit the activation of the Wnt/β-catenin pathway, while the elevation of TGFBR1 showed opposite effects.

**Conclusion:**

Tim-1 knockdown inhibited glioblastoma cell proliferation, invasion, and migration through the miR-133a/TGFBR1 axis and restrained the activation of the Wnt/β-catenin pathway.

**Supplementary Information:**

The online version contains supplementary material available at 10.1186/s12935-021-02036-1.

## Background

Glioblastoma, as a common and severe neurologic tumor, ranks the highest level in World Health Organization’s brain tumor classification [1, 2]. Glioblastoma is characterized by rapid dissemination, invasive growth, and high-degree cell heterogeneity [3]. The incidence of glioblastoma increases with age, and men are more susceptible to glioblastoma [4]. Glioblastoma typically progresses into a consequence of bad prognosis and poor life quality [5]. The common treatments (including surgery, radiotherapy, and chemotherapy) show no evident effect on the permanent therapy of glioblastoma [6]. Recently, a T cell immunity based-therapy has been reported as a promising method to enhance the prognosis of glioblastoma patients [7].

T-cell immunoglobulin and mucin domain 1 (Tim-1), as a crucial costimulatory molecule on T cell surface, is defined as a biomarker for T cell activation, which shows an essential role in modulating tumor-related immune response and promises to be a new therapeutic target for cancer therapy [8, 9]. Kong et al. [10]. demonstrated that TIM-1 can be regarded as a prognostic biomarker in gastric and lung adenocarcinoma through bioinformatics approaches, which is helpful to investigate the pathogenesis of their progression. TIM-1 is also been determined the frequently abnormal overexpression as a soluble form in CSF in patients with primary central nervous system lymphoma, served as a potential biomarker for PCNSL [11]. As has been evidenced previously, Tim-1 participates in the pathogenesis of multiple brain diseases. For example, Tim-1 expression is significantly elevated in middle cerebral artery occlusion/reperfusion, and silencing Tim-1 can alleviate the cerebral ischemia–reperfusion injury via reducing related cytokine production in brain tissues [12]. High expression of Tim-1 is associated with brain cancer stem cells [13]. Nevertheless, little has been reported about the role of Tim-1 in glioblastoma.

microRNAs (miRs), validated as short non-coding RNA molecules, are highly conserved, which modulates gene expression and diverse physiological processes in various cancers [14]. Recently, it has been suggested that miRs participate in the pathogenesis of glioblastoma, serving as regulators in glioma stem cell proliferation, apoptosis, and invasion [3]. miR-133 was firstly reported in mice and miR-133a downregulation could lead to an augmentation of contraction and bronchial smooth muscle (BSM) hyperresponsiveness. A previous study has also pointed out that miR-133a plays a protective role in glioblastoma via inhibiting the activation of MEK/PI3K/AKT and ERK pathways, showing great potential to be an effective therapy for glioblastoma [15]. Inhibition of TIM-3 has been found correlated with the reduced proliferation of leukemic stem cells in a miR-mediated way [16]. Nevertheless, no study has focused on the relationship between TIM -1 and miR-133a in glioblastoma, which is of great importance. Compared with the former biomarkers such as cytokines (IL-4, IL-13, and TGF-β) [17], Tim-1 is more than just a biomarker. Tim-1 has shown its great potential to become an immunotherapeutic target in the future with numerous questions to be further explored and confirmed, such as the crosstalk of Tim-1 in glioblastoma and immune cells in the immune microenvironment and the efficacy as biomarkers of glioblastoma cells. Therefore, this study aims to explore the mechanism of Tim-1 in regulating the proliferation, migration, and invasion of glioblastoma cells.

We hypothesized that Tim-1 played an underlying role in regulating glioblastoma through a miR-mRNA system. To verify this hypothesis, we first investigated that Tim-1 regulated TGFBR1 expression through miR-133a. Next, functional experiments were performed to testify the regulatory axis. Finally, the possible pathways were investigated. Investigating the possible Tim-1-miR-mRNA network may provide novel therapies against glioblastoma for clinical practice.

## Materials and methods

### Ethical statement

In the experiment, all tissue samples were sectioned and diagnosed by the pathology department of The Third Affiliated Hospital of Soochow University. The present study got approval from the Ethics Committee of The Third Affiliated Hospital of Soochow University.

### Sample collection

Glioblastoma samples collected from 76 patients undergoing surgical resection for the first time in The Third Affiliated Hospital of Soochow University were included as the experimental group. Another 15 normal brain tissues were collected as the control group (non-functional cortical brain tissues resected during the surgery for deep benign tumors). No significant difference in age and gender between patients was found in each group. The inclusion criteria were: (1) all patients admitted to hospital for the first time with no previous radiotherapy, chemotherapy, or immunotherapy before surgery; (2) all cases completed by the same group of neurosurgeons under the microscopy; (3) postoperative tumor tissues diagnosed as glioblastoma by pathology; (4) all cases with detailed and complete clinical data. Glioblastoma patients complicated with other tumors and metastatic tumors were excluded.

### Immunohistochemical (IHC) staining

Normal brain tissues and glioblastoma tissues were fixed in 4% paraformaldehyde, embedded in paraffin, and serially sectioned (3 μm). After dewaxing with Xylene, the sections were hydrated with different concentration of alcohol (100% alcohol: 10 min; 95% alcohol: 10 min; 80% alcohol: 10 min; and 70% alcohol: 10 min). After that, the sections were treated with Citric acid buffer (pH6.0) in microwave oven for antigen repair. The sections were incubated with 3% hydrogen peroxide solution in the dark and sealed using normal sheep serum at room temperature for 30 min. Afterwards, the sections underwent an overnight incubation at 4 °C with Tim-1 (10 µg/mL, Abcam, Cambridge, MA, USA), followed by the incubation with the horseradish peroxidase (HRP)-labeled secondary antibody (1:2000, Cell Signaling Technology, Beverly, MA, USA). Next, the sections were developed using 3,3’-diaminobenzidine working solution, and stained with hematoxylin. Each section was observed in different magnifications. According to the observation under a light microscopy (Leica optical instrument, Tokyo, Japan), the positive cells were those with brown-yellow granules in the cytoplasm or nucleus.

### Cells culturing

Human glioblastoma cell lines [U251 (Cell Bank of Type Culture Collection of the Chinese Academy of Sciences, Shanghai, China), U87, LN18 and A172 (FuHeng Cell Center, Shanghai, China)], and human astrocyte cell line HEB (GuangZhou Jennio Biotech Co., Ltd., Guangdong, China) were cultured in Dulbecco’s modified Eagles medium supplemented with 10% fetal bovine serum (FBS) (Gibco, Carlsbad, CA, USA) and penicillin/streptomycin [18].

### Cells grouping and transfection

Mixtures containing different vectors (25 µmol/L) were transfected into U251 and U87 cells with a firm compliance to the instructions of Lipofectamine™ 2000 kit (Invitrogen Inc., California, USA). Afterward, cells were incubated (5% CO_2_, 370 kit (Invitr U251 and U87 cells were allocated into the small harpin-negative control (sh-NC), sh-Tim-1, sh-Tim-1 + inhibitor NC, sh-Tim-1 + miR-133a inhibitor group. Next, they were allocated into the mimic NC, miR-133a mimic, miR-133a mimic + pcDNA3.1, and miR-133a mimic + pcDNA3.1-transforming growth factor beta (TGF-β) receptors type 1 (TGFBR1) groups (all from Suzhou GenePharma Co., Ltd., Suzhou, China). sh-Tim-1 could knockdown Tim-1 expression in cells relative to sh-NC. miR-133a inhibitor/mimic could downregulate/upregulate miR-133a expression in cells compared with inhibitor NC/mimic NC, respectively. pcDNA3.1-TGFBR1 could upregulate TGFBR1 expression in cells compared with pcDNA3.1 (Shanghai Solarbio Bioscience & Technology Co., Ltd.). The RNA sequences for transfection are as follows:

- shRNA-Tim-1: GCTCACCATTGTACTCTTACA.
- shRNA-NC: GAGTTCTCCGAAGGTGTCACG
- shRNA-Tim-1 Forward:
  GATCCGGGCTCACCATTGTACTCTTACAGGTACC
  TGTAAGAGTACAATGGTGAGCTTTTTG
- shRNA-Tim-1 Reverse: AATTCAAAAAGCTCACCATTGTACTCTTACAGGT
  ACCTGTAAGAGTACAATGGTGAGCCCG
- shRNA-NC Forward: GATCCGGGAGTTCTCCGAAGGTGTCACGCTCGAGCGTGAC
  ACCTTCGGAGAACTCTTTTTG
- shRNA-NC Reverse: AATTCAAAAAGAGTTCTCCGAAGGTGTCACGCT
  CGAGCGTGACACCTTCGGAGAACTC
- miR133a-3P mimics: UUUGGUCCCCUUCAACCAGCUG
- miR133a-3P inhibit: CAGCUGGUUGAAGGGGACCAAA
- miR133a-3P: NCCAGUACUUUUGUGUAGUACAA

### RNA-sequencing (RNA-Seq) analysis

The RNA-Seq analysis was entrusted by LC-Bio Technologies Co., Ltd (Hangzhou, China). The correlation analysis of miR and transcriptome can be divided into the following five steps: (1) target gene prediction of miR prediction; (2) correlation analysis of miR data and mRNA data; (3) correlation analysis of transcriptome data and gene data; (4) screening of significantly differentially expressed data according to the regulatory relationship; and (5) Gene Ontologies (GO) and KEGG enrichment analysis.

### Dual-luciferase reporter gene assay

Wild-type and mutant reporter gene fragments of TGFBR1 (Wt-TGFBR1, Mut-TGFBR1) containing miR-133a potential target sites were synthesized by GenePharma and cloned into psiCHECK-2 vectors (Invitrogen). The above vectors were co-transfected with miR mimic or miR NC, respectively, into U251 and U87 cells. After 48 h, a Dual Luciferase Reporter Assay Kit (Promega, Madison, WI, USA) was utilized for detecting the luciferase activity [19].

### Quantitative real time-polymerase chain reaction (qRT-PCR)

Total RNA of tissues and cells was extracted using TRIzol reagent (Ambion, Life Technologies Corp., Carlsbad, CA, USA) and reverse transcribed with PrimeScript RT kit (Promega). Tim-1, miR-133a and TGFBR1 were determined using SYBR Premier ExTaq (Takara Biotechnology Co., Ltd., Tokyo, Japan) and Mir-X miRNA First Strand Synthesis Kit (Takara), respectively. Glyceraldehyde-3-phosphate dehydrogenase (GAPDH) or U6 served as the standardized control for Tim-1, TGFBR1, and miR-133a. Gene relative mRNA expression was analyzed using the 2^−△△Ct^ method. Primer sequence is listed in Table 1.

**Table 1:** Primer sequence for qRT-PCR

Primer	Sequence (5'->3')
miR-133a	Forward primer: ACACTCCAGCTGGGTTTGTCCCCTTCAAC
	Reverse primer: TGGTGTCGTGGAGTCG
U6	Forward primer: CTCGCTTCGGCAGCACA
	Reverse primer: AACGCTTCACGAATTTGCGT
Tim-1	Forward primer: TACCCTGTATCAGGACCAGGA
	Reverse primer: GAGAGCTCTGTGCCTTCCAA
TGFBR1	Forward primer: GCAGAGCTGAGCCTTGAGAG
	Reverse primer: TGCCCTGTTGACTGAGTTGTG
GAPDH	Forward primer: CACCATCTTCCAGGAGCGAG
	Reverse primer: AAATGAGCCCCAGCCTTCTC

### Western blotting (WB)

Radioimmunoprecipitation assay buffer was utilized for cell lysis. Protein samples of an equal amount were subjected to 10% sodium dodecyl sulfate polyacrylamide gel electrophoresis for separation, which were then transferred to the polyvinylidene difluoride (PVDF) membranes (Millipore Corp., Billerica, MA, USA). Next, PVDF membranes underwent an overnight incubation at 4 °C with primary antibodies Tim-1 (4 μg/mL, ab47635), TGFBR1 (1:1000, ab235178), β-Catenin (1:5000, ab32572), c-myc (1:1000, ab32072), Cyclin D1 (1:200, ab16663), and GAPDH (1:1000, ab8245) (all from Abcam). After that, the HRP-labeled secondary antibody (1:2000, ab6721, Abcam) was added for a 2-h incubation at room temperature. Protein bands were detected by chemiluminescence. The target protein expression was calculated using ImageJ software (National Institutes of Health, Bethesda, MD, USA) and protein expression was standardized to GAPDH.

#### Cell Counting Kit-8 (CCK-8) detection

A CCK-8 kit (Dojindo Laboratories, Kumamoto, Japan) was utilized to observe cell proliferation as per the manufacturer’s instructions. Cells in logarithmic growth stage were seeded into 96-well microplates (1 × 10^4^ cells/well). CCK-8 (10 μL) was added to each well for a 4-h incubation at 37℃ at 0, 24, 48, and 72 h separately. The optical density (OD) value at 450 nm was measured using a microplate spectrometer (Bio-Rad Laboratories, Hercules, CA, USA).

#### Transwell assay

The Transwell chamber (8 μm pore; BD Biosciences, Bedford, MA, USA) was utilized for detecting cell invasion. Cells were cultured in a serum-free medium on the apical chamber pre-coated with matrigel (50 μL, BD Biosciences). The basolateral chamber was added with RPMI-1640 medium with 20% FBS. After 24 h, the invaded cells were fixed, stained with 1% crystal violet, and counted under the microscopy (Olympus Optical Co. Ltd., Tokyo, Japan) [19].

#### Wound healing test

Cells (5 × 10^5^ cells/well) were seeded into 6-well microplates. The bottom of 6-well microplates was covered the next day. The ruler was sterilized to make the vertical wound along the edge of the ruler using pipette tip (200 μL), with no inclination of the pipette tip. Each well was scratched three times in parallel. After that, the supernatant was discarded. Cells were washed 3 times using phosphate-buffered saline and added with serum-free medium to eliminate the influence of cell proliferation. Cells were photographed at 0 h and 24 h after scratch under the microscopy.

#### Statistical analysis

All data were analyzed using SPSS 22.0 (IBM Corp. Armonk, NY, USA). The results were expressed as mean ± standard deviation. Comparison between two groups was analyzed using the *t*-test. Comparison among groups was analyzed using one-way or two-way analysis of variance (ANOVA), and comparison between two groups was made followed by the t test with Bonferroni correction. Pearson was performed for correlation analysis. *p* < 0.05 is considered to be statistically significant.

## Results

### Tim-1 was upregulated in glioblastoma tissues and cells

**Fig. 1:**
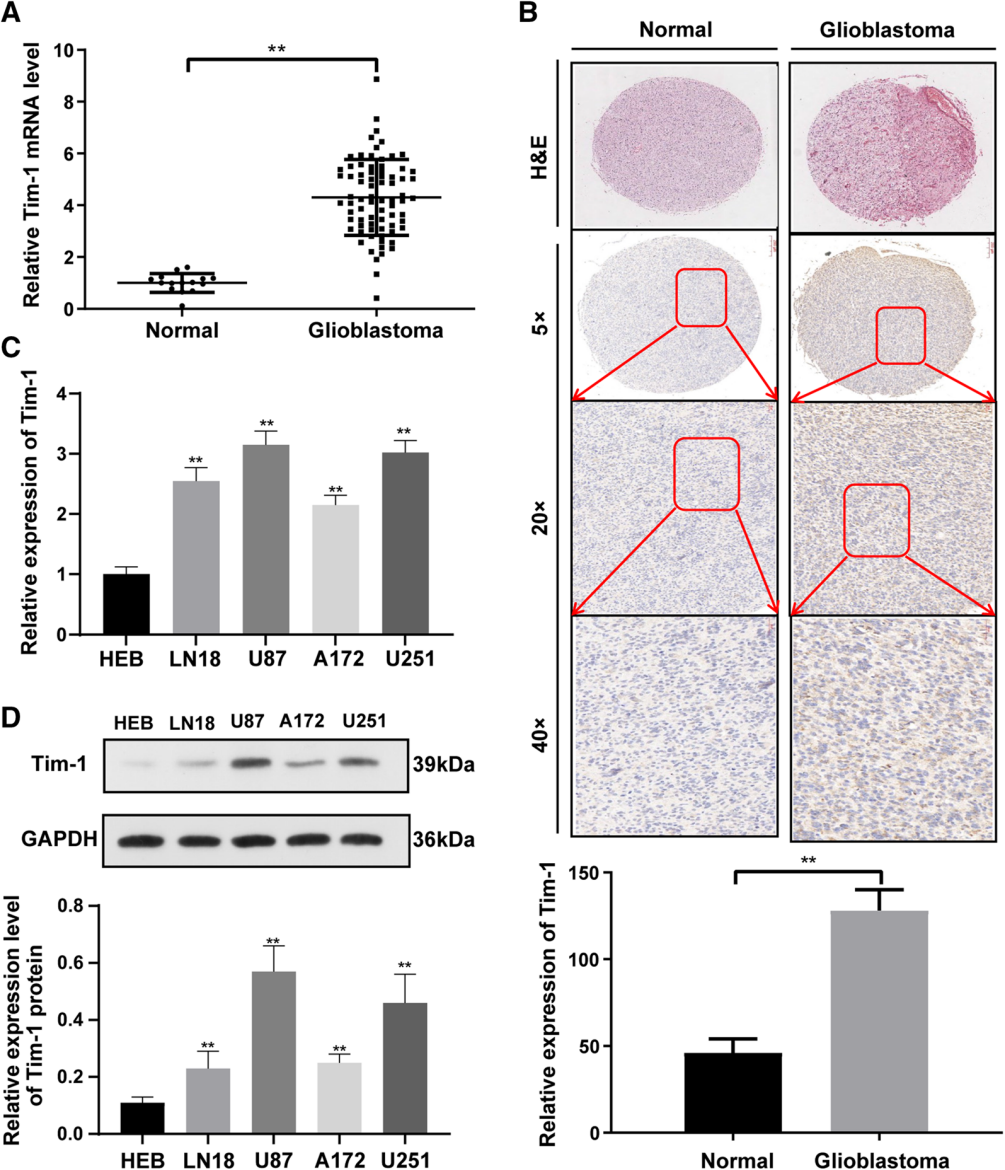
Tim-1 was upregulated in glioblastoma tissues and cells. **A** Tim-1 mRNA expression in normal brain tissues (n = 15) and glioblastoma tissues (n = 76) were detected using qRT-PCR. The relative mRNA levels of Tim-1 in different cells are compared with HEB cells which was identified as 1.0. **B** Differences in Tim-1 expression in normal brain tissues (n = 15) and glioblastoma tissues (n = 76) were detected using immunohistochemical staining (the cells stained by brown)and H&E staining(× 5, × 10, × 40); **C** Tim-1 expression in human astrocyte HEB and human glioblastoma cell lines was detected using qRT-PCR and WB (n = 3). The relative protein levels of Tim-1 in different cells are compared with HEB cells which was identified as 1.0.* represented comparison with HEB, *p* < 0.05. Data were expressed as mean ± standard deviation, and *t*-test was used for comparisons between two groups. One-way ANOVA was used for comparisons between multiple groups, followed by t test with Bonferroni correction

According to the results of qRT-PCR (Fig. 1A, P < 0.01) and IHC staining (Fig. 1B, p < 0.05), Tim-1 mRNA expression and protein level were all significantly higher in glioblastoma tissues compared with those in normal brain tissues. Tim-1 expression was higher in human glioblastoma cell lines than that in human astrocyte HEB, and U87 and U251 cells with the highest Tim-1 expression were selected for the subsequent experiments (Fig. 1C, all *p* < 0.05). Moreover, the cell density of glioblastoma tissues was elevated with the increase of Tim-1 expression, which suggested that high Tim-1 expression may be negatively correlated with glioblastoma malignant proliferation.

### Tim-1 regulated the miR-133a/TGFBR1 axis

**Fig. 2:**
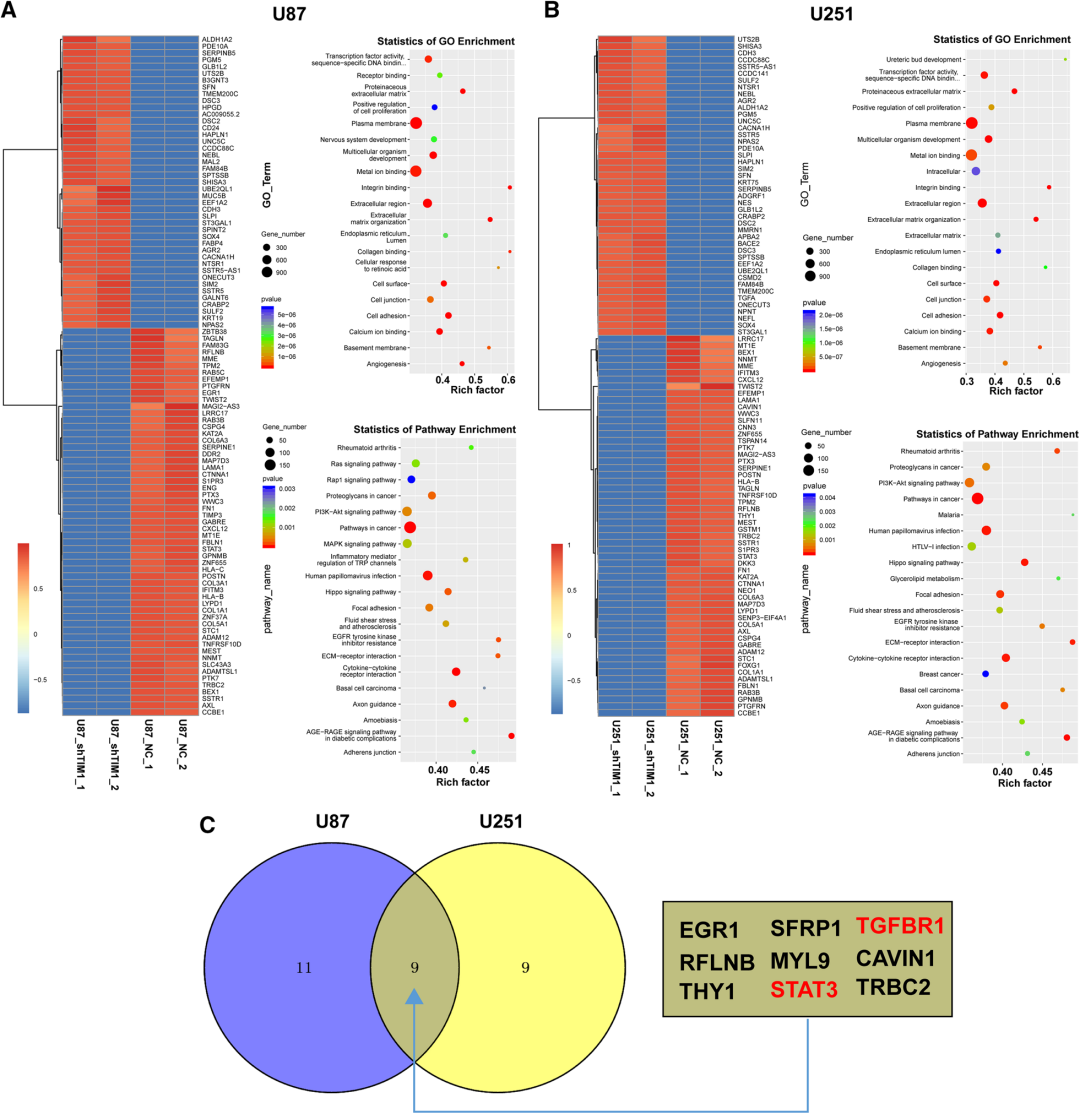
Tim-1 regulated the expression of TGFBR1. Genes with significantly differential expression in U87 and U251 (**A**) cells after Tim-1 downregulation; the scatter diagram of GO function enrichment analysis results in U87 and U251 (**B**) cells after Tim-1 downregulation; the scatter diagram of KEGG enrichment analysis results in U87 (**A**) and U251 (**B**) cells after Tim-1 downregulation; Genes with significantly differential expression both in U87 and U251 cells (**C**)

**Fig. 3:**
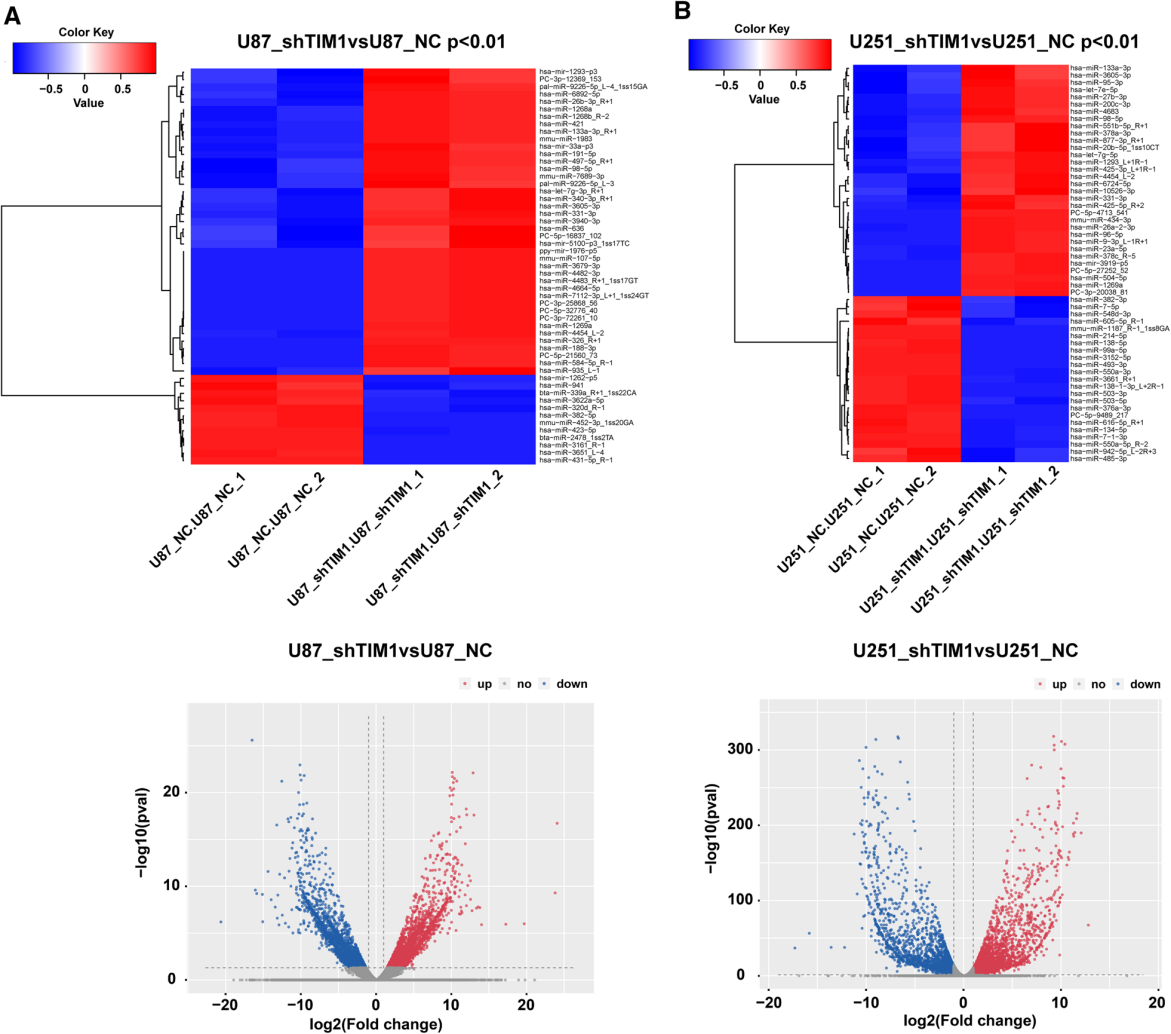
miRs with significantly differential expression and their corresponding heat map in U87 (**A**) and U251 (**B**) cells after Tim-1 downregulation

To identify the downstream mechanism of Tim-1, the expressions of genes were analyzed through RNA-seq analysis after the downregulation of Tim-1 in U87 and U251 cell lines; the differential pathways were analyzed by bioinformatics (Fig. 2A, B). Nine genes (EGR1, SFRP1, TGFBR1, RFLNB, MYL9, CAVIN1, THY1, STAT3, and TRBC2) (Fig. 2C) showed obvious correlations with Tim-1 knockdown, which also indicated the correlation between the abnormally expressed Tim-1 and TGFBR1 in glioma. To explore the regulation of Tim-1 gene in TGFBR1 expression, microRNA RNA-seq was performed; it was found that miR-133a was remarkably increased in U87 and U251 cell lines after Tim-1 knockdown (Fig. 3A, B).

**Fig. 4:**
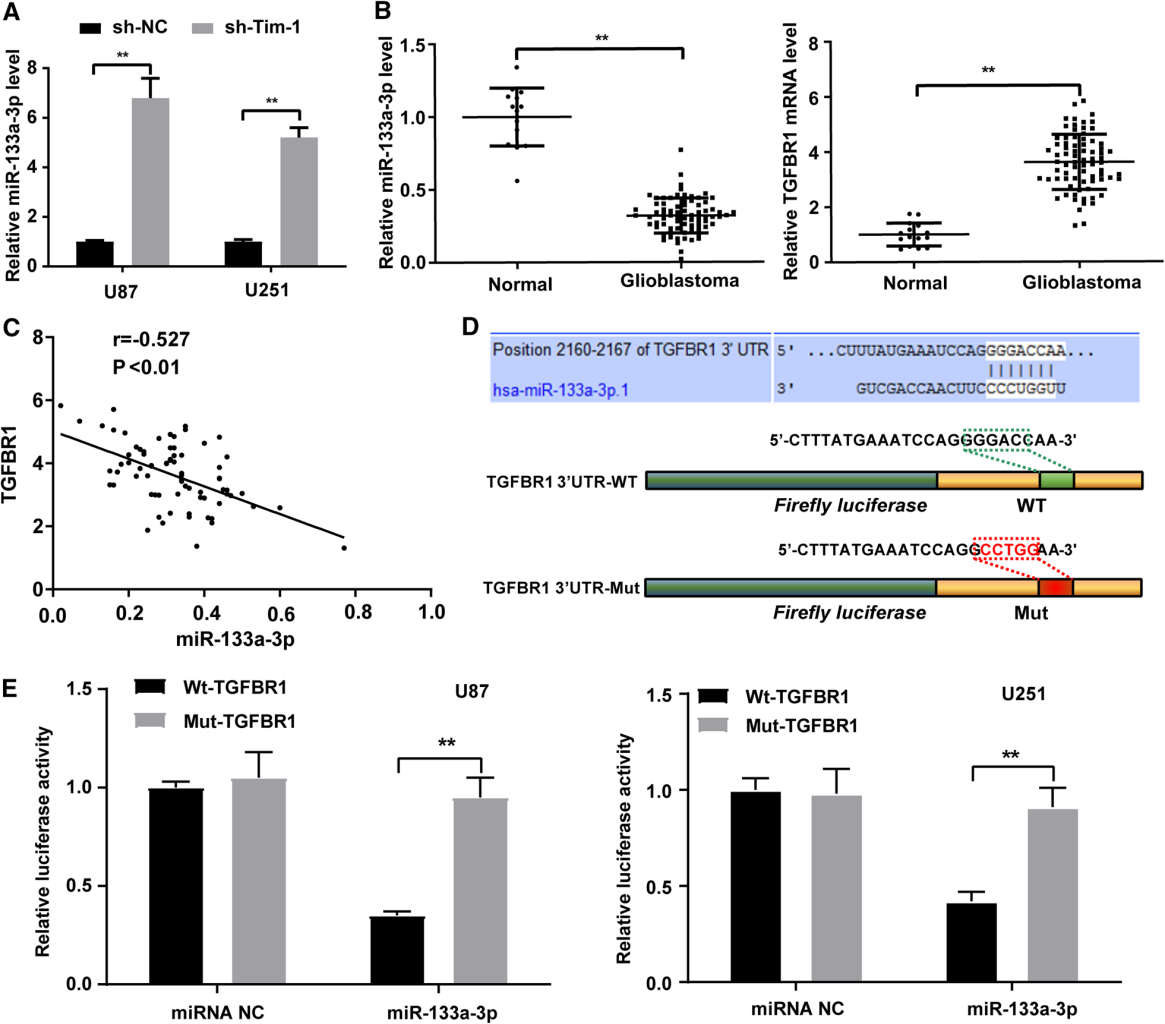
Tim-1 regulated the expression of miR-133a. **A** miR-133a expression was significantly increased in U87 and U251 cell lines after Tim-1 knockdown (n = 3); **B** miR-133a and TGFBR1 mRNA level in normal brain tissues (n = 15) and glioblastoma tissues (n = 76) were detected using qRT-PCR; **C** Pearson-Correlation analysis of miR-133a and TGFBR1 in human glioblastoma tissues; **D** Construction of Wt and Mut vectors of TGFBR1-3’UTR; N. The luciferase activity U87 and U251 cells co-transfected with miR-133a mimic or miR NC and Wt-TGFBR1 or Mut-TGFBR1 (n = 3). Data were expressed as mean ± standard deviation, and *t*-test was used for comparisons between two groups. One-way ANOVA was used for comparisons between multiple groups, comparison between two groups are followed by t test with Bonferroni correction

Next, miR-133a expression was detected in glioblastoma cells U87 and U251 with the intervention of Tim-1 knockdown. According to the results, miR-133a was notably upregulated after Tim-1 knockdown (Fig. 4A). qRT-PCR results revealed that miR-133a expression in glioblastoma was clearly decreased, with the increased expression of TGFBR1 (Fig. 4B). Pearson’s correlation analysis revealed that miR-133a was negatively correlated with TGFBR1 expression (*r* = − 0.527, *p* < 0.01) (Fig. 4C). Meanwhile, through the bioinformatics (http://www.targetscan.org/vert_72/), we identified TGFBR1 as a predicted target of miR-133a. There was a specific binding region between the TGFBR1 gene sequence and miR-133a sequence (Fig. 4D), which was also verified by dual-luciferase reporter gene assay. As shown in Fig. 4E, the luciferase activity of Wt-TGFBR1 3’UTR was dramatically reduced after transfection with miR-133a mimic, while that of Mut-TGFBR1 reversed no reduction. From all above, we confirmed that TGFBR1 was a target of miR-133a, and Tim-1 regulated miR-133a expression.

### Tim-1 regulated glioblastoma cell proliferation, invasion, and migration by miR-133a

**Fig. 5:**
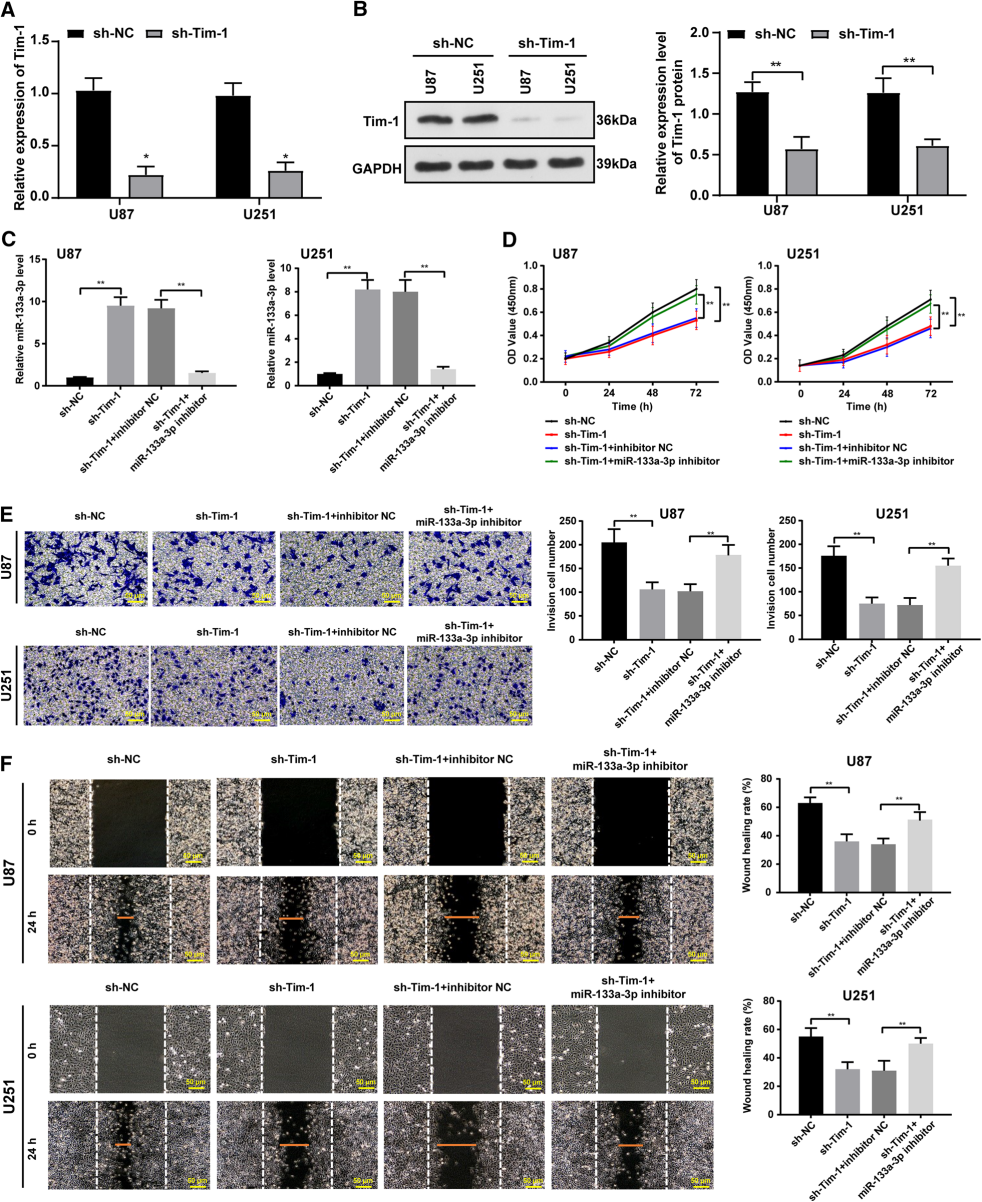
Downregulated Tim-1 inhibited the proliferation, invasion and migration of glioblastoma cells, which could be reversed by downregulating miR-133a. **A** The expression of Tim-1 in U87 and U251 cells with Tim-1 knock-down were detected using qRT-PCR and WB; **B** The expression of miR-133a in U87 and U251 cells was detected using qRT-PCR; **C** The proliferation ability of U87 and U251 cells was detected by CCK-8 detection; **D** The invasion ability of U87 and U251 cells was detected by Transwell assay; **E** The migration ability of U87 and U251 cells was detected by wound healing test. *represented comparison with sh-NC, *p* < 0.05; + represented comparison with sh-Tim-1 + inhibitor NC, *p* < 0.05. n = 3. Data were expressed as mean ± standard deviation, and *t*-test was used for comparisons between two groups. One-way ANOVA was used for comparisons between multiple groups, comparison between two groups are followed by t test with Bonferroni correction

Tim-1 expression in U87 and U251 cells was firstly knocked down, which was confirmed using qRT-PCR and WB (Fig. 5A). Next, the inhibition ability of miR-133a inhibitor was testified. As presented by qRT-PCR results, Tim-1 downregulation could notably enhance miR-133a expression, which was then decreased after miR-133a inhibition (all *p* < 0.05) (Fig. 5C).

CCK-8 detection (Fig. 5D), Transwell assay (Fig. 5E), and wound healing test (Fig. 5F) were used to verify the effect of downregulated Tim-1 combined with miR-133a inhibitor on the biological behaviors of glioblastoma cells. The results showed that downregulation of Tim-1 inhibited glioblastoma cell proliferation, invasion, and migration, while miR-133a inhibition reversed the above effects (all *p* < 0.05).

### Upregulated miR-133a inhibited glioblastoma cell biological behaviors via targeting TGFBR1

**Fig. 6:**
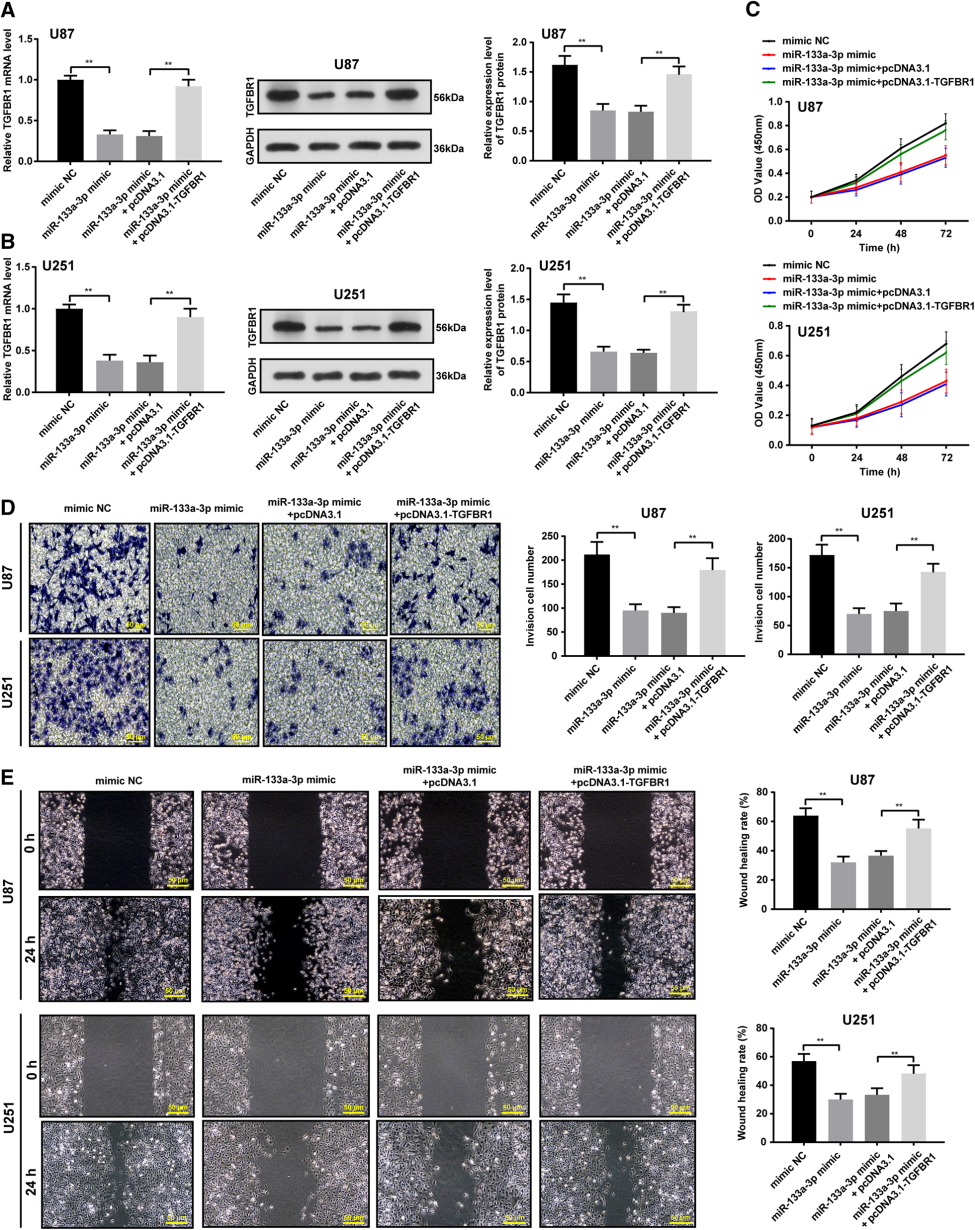
Upregulated miR-133a inhibited the proliferation, invasion and migration of glioblastoma cells, which could be reversed by upregulated TGFBR1. TGFBR1 expression in U87 and U251 cells was detected using qRT-PCR (**A**) and WB (**B**); **C** The proliferation ability of U87 and U251 cells was detected by CCK-8 detection; **D** The invasion ability of U87 and U251 cells was detected by Transwell assay; **E** The migration ability of U87 and U251 cells was detected by wound healing test. *represented comparison with mimic NC, *p* < 0.05; + represented comparison with miR-133a mimic + pcDNA3.1, *p* < 0.05. n = 3. Data were expressed as mean ± standard deviation. One-way ANOVA was used for comparisons between multiple groups, comparison between two groups are followed by t test with Bonferroni correction

Subsequently, we elevated miR-133a expression with miR-133p mimic (Fig. 6A, B), and the changes of TGFBR1 expression were detected in glioblastoma cells U87 and U251 using qRT-PCR and WB. The results demonstrated that miR-133a upregulation obviously reduced TGFBR1 expression, which was reversed after the treatment of pcDNA3.1-TGFBR1 (all *p* < 0.05).

CCK-8 detection (Fig. 6C), Transwell assay (Fig. 6D), and wound healing test (Fig. 6E) were used to verify the effect of upregulated miR-133a combined with the upregulation of TGFBR1 on glioblastoma cells. The results showed that miR-133a downregulation inhibited glioblastoma cell proliferation, invasion, and migration, while TGFBR1 upregulation reversed the above effects (all *p* < 0.05).

### Effects of downregulated Tim-1 or upregulated miR-133a on the Wnt/β-catenin pathway in glioblastoma cells

**Fig. 7:**
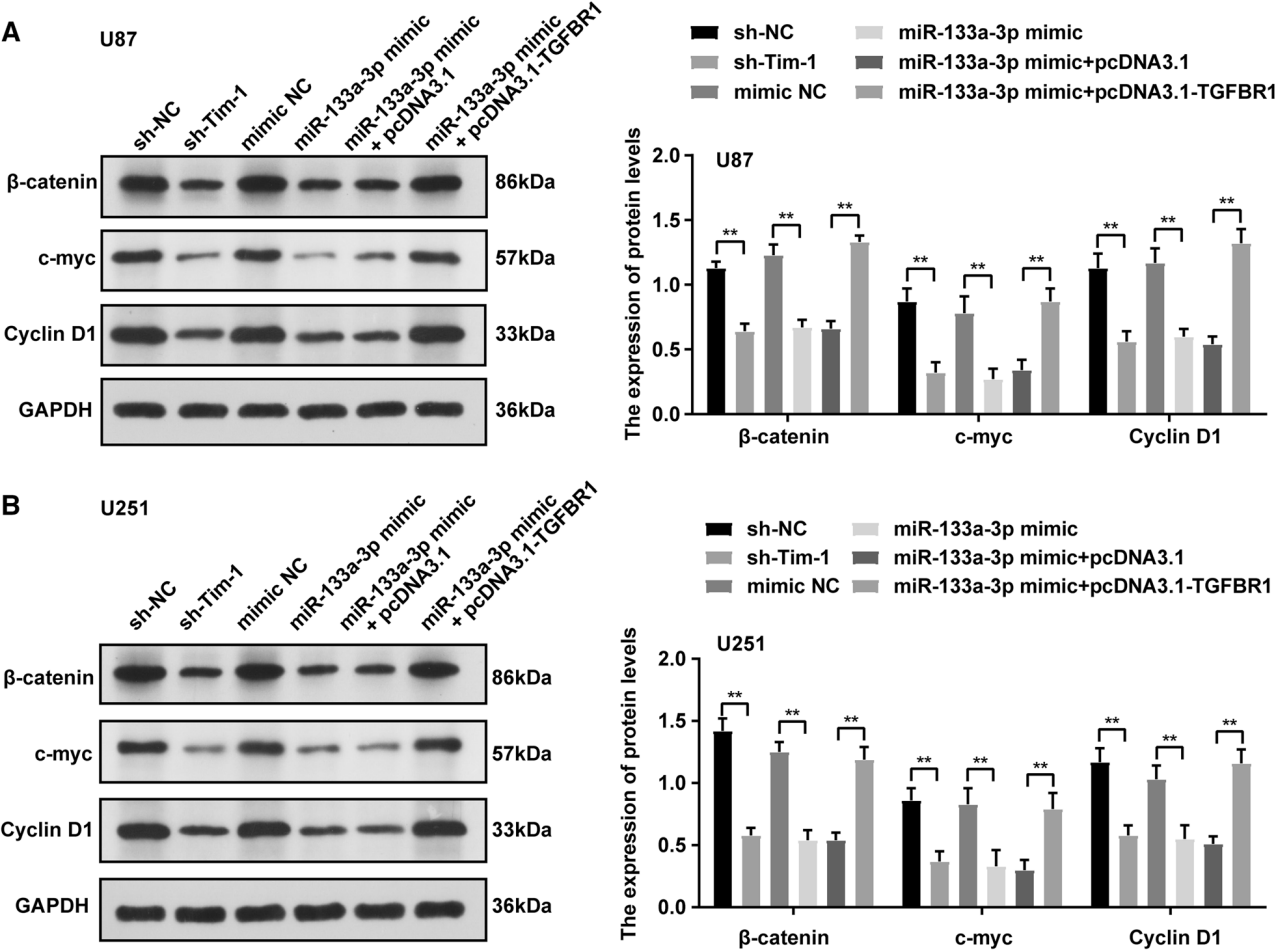
Effects of downregulated Tim-1 or upregulated miR-133a on Wnt/β-catenin signaling pathway in glioblastoma cells. Wnt/β-catenin-related β-catenin, c-myc and Cyclin D1 proteins levels in U87 (**A**) and U251 (**B**) cells were detected using WB. *represented comparison with sh-NC, *p* < 0.05; + represented comparison with mimic NC, *p* < 0.05; # represented comparison with miR-133a mimic + pcDNA3.1, *p* < 0.05. n = 3. Data were expressed as mean ± standard deviation. One-way ANOVA was used for comparisons between multiple groups, comparison between two groups are followed by t test with Bonferroni correction

Finally, the possible pathways involving in this process were investigated. As has been evidenced previously, the Wnt/β-catenin pathway mediates the proliferation of glioblastoma cells [20]. TGF-β (TGFBR1 is TGF-β receptor 1) can induce the upregulation of Wnt3 and the β-catenin pathway in breast cancer cells [21]. Therefore, effects of Tim-1 and miR-133a on the Wnt/β-catenin pathway in glioblastoma cells were evaluated using WB (Fig. 7A, B). It was observed that downregulated Tim-1 or upregulated miR-133a could suppress the activation of the Wnt/β-catenin pathway, as manifested by the downregulation of c-myc, β-catenin, and Cyclin D1 in U87 and U251 cells, while TGFBR1 upregulation could reverse the above inhibitory regulation of miR-133a in the Wnt/β-catenin pathway.

## Discussion

Glioblastoma, as a lethal human tumor with extremely low survival rates, paralyzes anti-tumor immunity and causes severe T cell dysfunction [22, 23]. It has been suggested that Tim-1 regulates T cell activation and plays vital roles in modulating various autoimmune diseases [24]. Recent clinical studies [25] have demonstrated the feasibility and safety of CAR T-cell therapy for glioblastoma. It is reported [26] that the neoantigen-specific T cells from the peripheral blood can migrate into an intracranial glioblastoma tumor, thus neoantigen-targeting vaccines may have the role to favorably alter the immune microenvironment of glioblastoma. However, the underlying mechanism under glioblastoma is still elusive. In this study, we identified that Tim-1 was abnormally elevated in glioblastoma, and its knockdown inhibited glioblastoma cell proliferation, invasion, and migration via the miR-133a/TGFBR1 axis and the Wnt/β-catenin pathway activation (Additional file 1: Figure S1).

T cells are involved in altering the tumor immune microenvironment to play the antitumor function. During this process, the mucin domain protein-1 (TIM-1), as a costimulatory molecule on the surface of T cells, has a strong regulatory effect on T cells [8]. TIM-1 could accelerate the type 1 immune response in solid tumors. It has been reported that Tim-1 is aberrantly expressed in human cancers, showing a potential influence on cancer progression [9]. CD19^+^ TIM‐1^+^ cells are reported to promote tumor growth, which could be alleviated via a miR-mediated regulation [27]. In this study, we identified that Tim-1 participated in the pathogenesis of glioblastoma via regulating the miR-133a/TGFBR1 axis. A previous study has shown that miR-133a is downregulated in colorectal cancer [28]. miR-133a plays vital roles in promoting glioblastoma cells’ resistance to recombinant tumor necrosis factor-related apoptosis-inducing ligand [29]. Moreover, it has provided evidence that miR-133a targets TGFBR1 [30]. TGFBR1, as a key TGF-β receptor, is involved in various biological processes [31]. Tim-1^+^ B cells are associated with significantly elevated TGF-β1 [23]. Deregulated TGFBR1 is tightly associated with glioblastoma progression [33].

Furthermore, our study also revealed that downregulated Tim-1 or upregulated miR-133a inhibited glioblastoma cell biological behaviors. In a prior work, Tim-1 regulates the cellular activity of cancer cells, and Tim-1 knockdown significantly diminishes non-small-cell lung carcinoma cell migration and invasion [9]. Overexpressed miR-133a-3p suppresses the proliferation, invasion, and mitosis, as well as the migration of oral squamous cell carcinoma cells [34]. Besides, the overexpression of miR-133a-3p can suppress fibrosis by down-regulating IRF5 and then inhibiting the TGF-β/Smad2 signaling pathway, therefore, promoting apoptosis and reducing the proliferation in keloid fibroblasts [35]. Taken together, silencing Tim-1 could inhibit glioblastoma cell growth via regulating the miR-133a/TGFBR1 axis.

β-catenin, Cyclin D1, and c-myc are critical transcription factors and target genes of the Wnt/β-catenin pathway, which be used as biomarkers for evaluating the Wnt/β-catenin pathway activation [36]. Li et al. [37]. demonstrated the important roles of WNT/β-catenin signaling in the T cell-inflamed and non-T cell-inflamed tumor microenvironments, such as the immune escape, development and function of immune cells, and immunosurveillance. As shown by our results, expressions of β-catenin, Cyclin D1, and c-myc were notably reduced after Tim-1 knockdown or miR-133a upregulation, which could be reversed by TGFBR1 upregulation, which indicated that Tim-1 knockdown inhibited the Wnt/β-catenin pathway activation via the miR-133a/TGFBR1 axis in glioblastoma. As has been evidenced previously, the abnormal activation of the Wnt/β-catenin pathway is closely related to tumor proliferation and invasion in glioblastoma [38]. miR-133a inhibit ovarian cancer cell proliferation, invasion, and migration, with the involvement of Wnt/β-catenin pathway [39]. TGF-β induces Wnt/β-catenin signaling activation [21]. Briefly, we proved that Tim-1 knockdown suppressed the Wnt/β-catenin pathway activation via the miR-133a/TGFBR1 axis in glioblastoma, and it promise to be a potential drug target in clinical practice. In previous study, Tim-1 initially functions as a crucial costimulatory molecule on T cell surface, and we are the first study to analyze the important role of Tim-1 in regulating the proliferation, migration, and invasion of glioblastoma cells via the miR-133a/TGFBR1 axis and the Wnt- β catenin pathway.

## Conclusion

In conclusion, this study supported that Tim-1 knockdown inhibited glioblastoma cell biological behaviors and the Wnt/β-catenin pathway activation via regulating the miR-133a/TGFBR1 axis. Blocking the Tim-1/miR-133a/TGFBR1 axis might develop as a novel robust therapeutic approach for glioblastoma treatment.

## Supplementary Information

**Additional file 1: Figure S1. ** Mechanism diagram: Tim-1 knockdown upregulated miR-133a expression, and miR-133a targeted TGFBR1, then inhibited the activation of Wnt/β-catenin pathway, thereby inhibiting the proliferation, invasion and migration of glioblastoma cells.

## Data Availability

Not applicable.

## Abbreviations

Tim-1: T-cell immunoglobulin and mucin domain 1
miR: MicroRNA
TGFBR1: Transforming growth factor beta receptors type 1
NC: Negative control
sh-NC: Small harpin-negative control
FBS: Fetal bovine serum
HRP: Horseradish peroxidase
GO: Gene ontologies
RNA-Seq: RNA-sequencing
KEGG: Kyoto Encyclopedia of Genes and Genomes
Wt: Wild-type
Mut: Mutant
GAPDH: Glyceraldehyde-3-phosphate dehydrogenase
CCK-8: Cell counting Kit-8
ANOVA: Analysis of variance
