# Supplementary figures and images for: Downregulation of Tim-1 inhibits the proliferation, migration and invasion of glioblastoma cells via the miR-133a/TGFBR1 axis and the restriction of Wnt/β-catenin pathway
Source: Cancer Cell Int. 2021 Jul 5;21:347. doi: 10.1186/s12935-021-02036-1 (PMC8256541; doi:10.1186/s12935-021-02036-1)

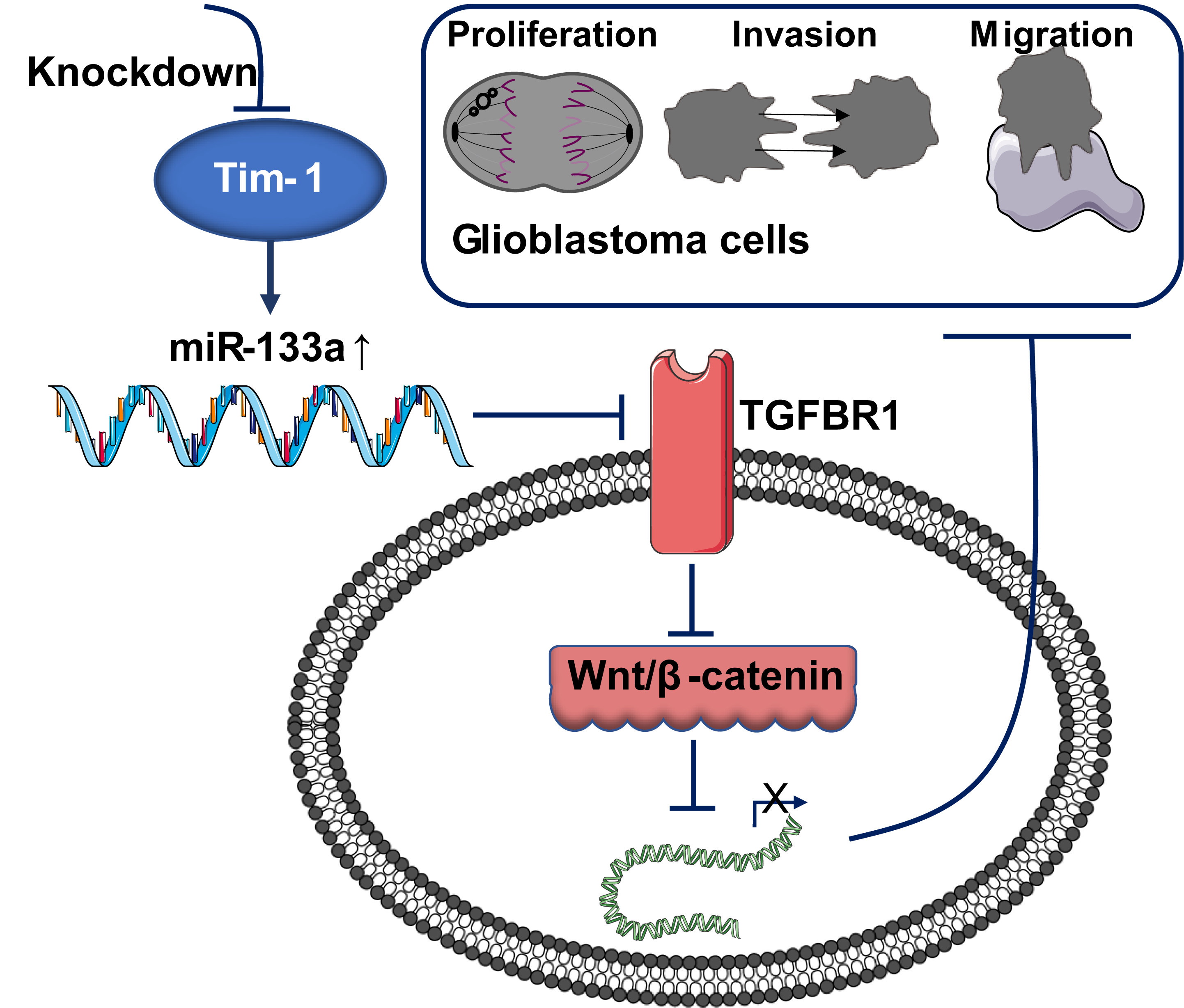

Supplement: Supplementary file 1 — Additional file 1: Figure S1. Mechanism diagram: Tim-1 knockdown upregulated miR-133a expression, and miR-133a targeted TGFBR1, then inhibited the activation of Wnt/β-catenin pathway, thereby inhibiting the proliferation, invasion and migration of glioblastoma cells. [file 12935_2021_2036_MOESM1_ESM.tif]
